# Case Report: A case of subacute combined degeneration of the spinal cord associated with chronic atrophic gastritis, macrocytic anemia, and positive anti-sulfatide IgM and IgG antibodies

**DOI:** 10.3389/fimmu.2025.1582265

**Published:** 2025-05-05

**Authors:** Shun-Zhi Zhuang, Cai-ming Li, Ling-en Kong, Bi-hua Lin, Cai-ling Zhu

**Affiliations:** ^1^ Department of Neurology, Huizhou First Hospital, Huizhou, China; ^2^ Department of Radiology, Huizhou First Hospital, Huizhou, China; ^3^ Department of Critical Care Medicine, Huizhou First Hospital, Huizhou, China

**Keywords:** subacute combined degeneration of the spinal cord, anti-sulfatide antibodies, chronic atrophic gastritis, macrocytic anemia, case

## Abstract

**Background:**

Anti-sulfatide antibodies are autoantibodies that are associated with various neurological disorders. They have been identified in several conditions including Guillain–Barré syndrome, multiple sclerosis, and chronic inflammatory demyelinating polyneuropathy. Subacute combined degeneration of the spinal cord is mainly caused by vitamin B12 deficiency. Chronic atrophic gastritis is a complex condition characterized by atrophy and inflammation of the gastric mucosa. To date, there have been no reported cases of patients with subacute combined degeneration of the spinal cord co-occurring with chronic atrophic gastritis and positive anti-sulfatide antibodies.

**Case presentation:**

A 58-year-old male patient presented with symmetrical progressive numbness of his limbs. Although he did not experience gait instability, he had difficulty performing fine motor tasks in his upper limbs. We reviewed the patient’s medical history and analyzed the results of his blood tests, imaging studies, and gastroscopic. The patient met the criteria for subacute combined degeneration of the spinal cord, chronic atrophic gastritis, macrocytic anemia, and anti-sulfatide antibody positivity. His symptoms improved after treatment.

**Conclusion:**

This is the first report on the coexistence of positive anti-sulfatide antibodies and subacute combined degeneration of the spinal cord, including chronic atrophic gastritis and macrocytic anemia, which will enhance our understanding of the relationships between these conditions.

## 1 Introduction

Chronic atrophic gastritis (CAG) is a common chronic digestive system disorder, characterized primarily by abdominal pain, bloating, and discomfort. Some patients may experience numbness and symptoms associated with other neurological disorders. Its pathological features include infiltration of inflammatory cells and atrophy of the gastric mucosal glands, particularly reduction or disappearance of parietal cells (1, 2). Moreover, intrinsic factors such as insufficient secretion of certain substances can impair the absorption of vitamin B12 (VitB12) (3), leading to its deficiency in the body. This deficiency can damage neurons and subsequently cause peripheral neuropathy (4). Subacute combined degeneration (SCD) of the spinal cord is a neurodegenerative disorder primarily caused by VitB12 deficiency (4). It results from both dietary and non-dietary causes and is typically secondary to malnutrition syndromes such as chronic alcoholism, strict vegetarianism, gastrectomy, and nitrous oxide abuse. Sulfatide is a prevalent glycolipid in the spinal cord and peripheral nerves and is particularly concentrated in peripheral nerve myelin. Anti-sulfatide antibodies have been found in several diseases. A study involving 25 patients with elevated sulfatide antibodies found that 98% had peripheral neuropathy. Most patients with these antibodies experience pure sensory neuropathy rather than sensorimotor neuropathy (5). To our knowledge, there have been no reported cases of coexistence of CAG, spinal cord SCD, and positive anti-sulfatide antibodies. We report a case of anti-sulfatide antibody positivity associated with spinal cord SCD, CAG, and macrocytic anemia and discuss the potential connections between these conditions.

## 2 Case presentation

A 58-year-old male patient was admitted to our hospital with a 10-year history of numbness in both feet, which had worsened over the past six months with the addition of numbness in both upper limbs. The patient had developed numbness in both feet without any obvious cause 10 years previously, accompanied by lower back pain. The patient did not experience any limb weakness or gait instability. The condition had not been treated and had worsened over the past six months, with numbness extending below the hip joints. He gradually developed distal numbness in both upper limbs with poor coordination in the upper limbs, which manifested as difficulty in writing and using chopsticks. The patient also experienced constipation and difficulty urinating. Throughout the course of illness, the patient did not experience dizziness, neck pain, or gait instability. He denied any history of chronic diseases, such as diabetes, kidney disease, or rheumatic immune disorders. He had a 15-year history of alcohol consumption, predominantly white liquor (50 ml/day, 40% alcohol by volume) consumption. Upon admission, neurological examination revealed normal orientation, mental status, muscle strength (grade 5 in all four limbs), coordination, and gait. Tendon reflexes were diminished in the upper limbs and absent in the lower limbs, with no pathological signs elicited. Diminished superficial sensation was noted below the wrists in the upper limbs and below the hips in the lower limbs.

Laboratory tests revealed macrocytic anemia (**Table 1**): decreased red blood cell (RBC) count, decreased hemoglobin (Hb), elevated mean corpuscular volume (MCV), and elevated mean corpuscular hemoglobin (MCH). Serum vitamin B12 (195 pg/mL, normal range 133 pg/mL–675 pg/mL) and folate (17.99 ng/mL, normal range >5.38 ng/mL) levels were within normal limits. Thyroid function tests showed elevated TSH (8.342 mIU/L, normal range 0.55 mIU/L–4.78 mIU/L), while free T3 (FT3) and free T4 (FT4) levels were within normal limits. Liver function tests, plasma electrolytes, antinuclear antibody profile, and syphilis serology were all within normal limits. Lumbar puncture revealed normal intracranial pressure (110 mmH_2_O) and normal routine and biochemical results in the cerebrospinal fluid (CSF).

**Table 1:** Laboratory test results before and after treatment.

Items	Measured value				Reference range
	Before treatment	After 2 weeks of treatment	After 4 weeks of treatment	After 5 weeks of treatment	
Hb (g/L)	123	143	140	139	130–175
RBC (×10^12/L)	3.38	4.05	4.19	4.29	4.30–5.80
HCT (L/L)	0.368	0.415	0.427	0.415	0.400-0.500
MCV (fL)	108.9	102.5	101.9	96.7	82.0–100.0
MCH (Pg)	36.4	35.3	33.4	32.4	27.0–34.0
MCHC (g/L)	334	345	328	335	316-354

Hb, hemoglobin; RBC, red-cell count; HCT, hematocrit; MCV, mean corpuscular volume; MCH, mean corpuscular hemoglobin; MCHC, mean corpusular hemoglobin concentration.

Neurophysiological testing revealed the absence of sensory nerve conduction velocity (SCV) in the bilateral common peroneal nerves, suggesting severe mixed damage in the sensory fibers. The left median nerve showed a slowed SCV with a low-normal amplitude, while the right median nerve had a normal SCV but a low amplitude. The left ulnar nerve exhibited a slowed SCV with normal amplitude, and the right ulnar nerve had normal SCV and amplitude. Electromyography (EMG) of the left abductor pollicis brevis, abductor digiti minimi, extensor digitorum, and biceps brachii muscles showed no denervation potentials at rest. During minimal contraction, motor unit potentials were slightly prolonged with increased amplitude and normal polyphasic waves, and mixed potentials were observed during maximal contraction, suggesting possible neurogenic damage at the C5-T1 level on the left side. The bilateral upper limb somatosensory evoked potential (SEP) was abnormal, suggesting possible cervical segmental involvement of the sensory nerve conduction pathways, which was more pronounced on the left side.

**Figure 1:**
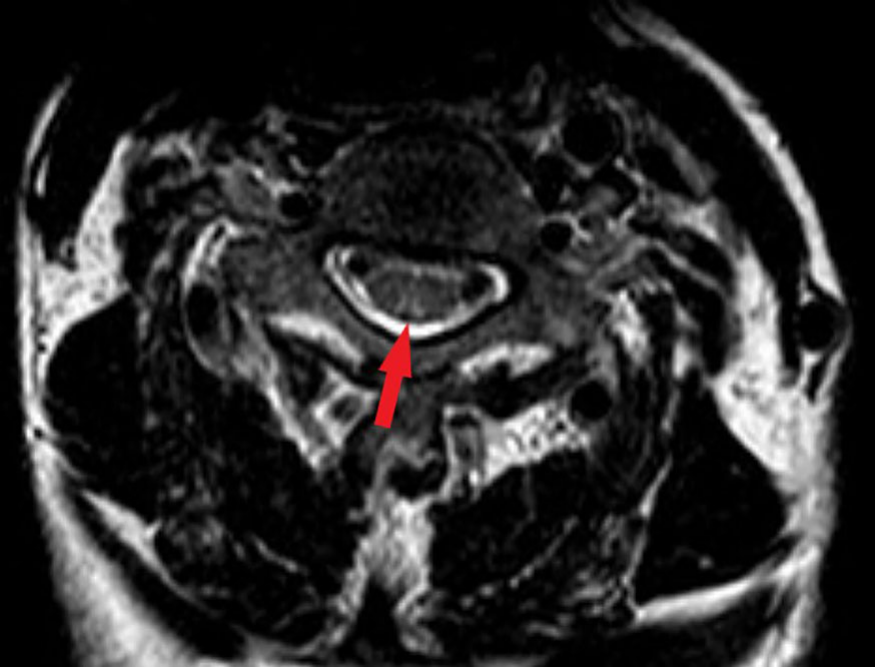
Magnetic resonance image (MRI) findings in the spinal cord. The T2-weighted image showed bilateral corticospinal tracts inverted V or “rabbit ears” sign on cervical spinal (red arrow).

**Figure 2:**
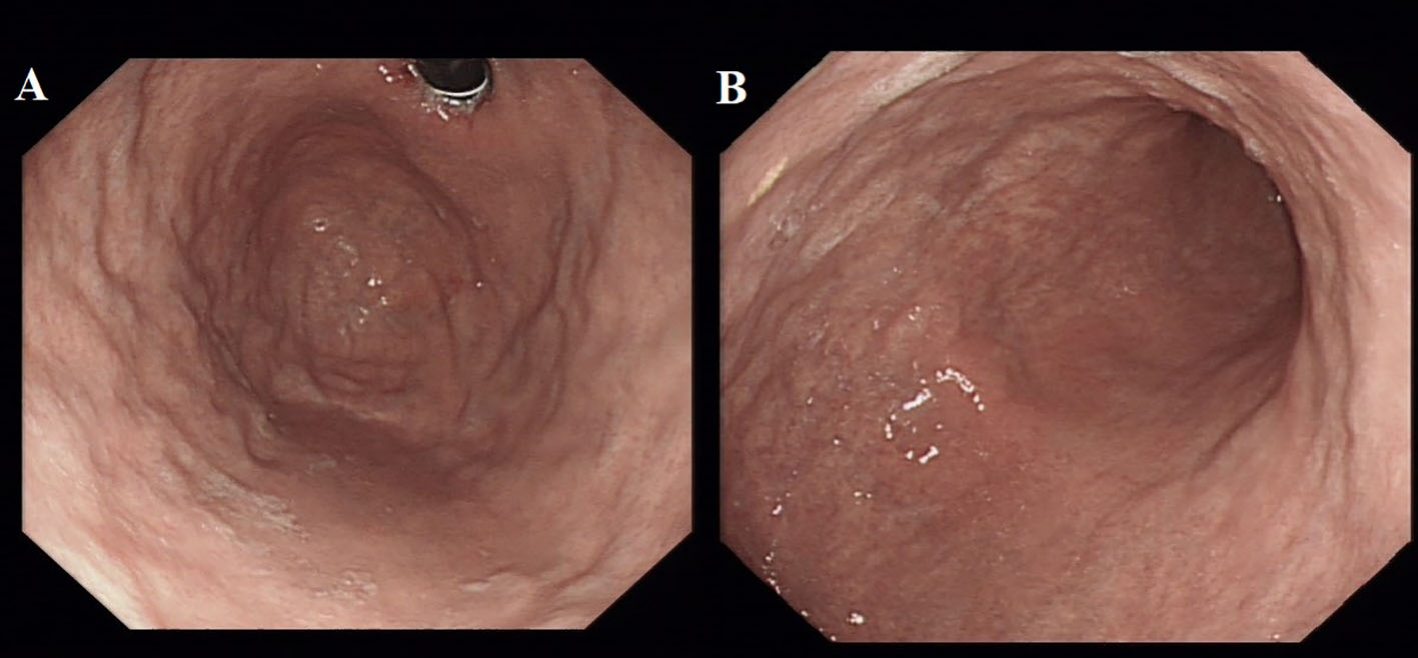
Gastroscope shows the atrophic gastritis in fundus **(A)** and corpus **(B)**.

Spinal magnetic resonance imaging (MRI) revealed linear high-signal foci on T2-weighted imaging (T2WI) in the posterior columns of the cervical spinal cord, presenting as an inverted “V” or “rabbit ears” sign, symmetric bilaterally and with a wide distribution (**Figure 1**). Gastroscopy revealed atrophic gastritis in the gastric body and fundus, with mild congestion and exudative gastritis in the antrum (**Figure 2**). The cardia showed focal, rough, congested, and eroded mucosa. Histopathological examination revealed mild chronic inflammation with erosion and focal intestinal metaplasia of the mucosa, with active inflammation.

Based on all the examination results, the patient was diagnosed with 1) spinal cord SCD, 2) CAG, and 3) macrocytic anemia. The patient showed no significant improvement after one week of VitB12 intramuscular injections (0.5 mg/d). Given the severe peripheral nerve involvement, immune-mediated peripheral neuropathy was considered. Further testing revealed the presence of anti-sulfatide IgM and IgG antibodies in serum. Based on these findings, the patient was diagnosed with immune-related peripheral neuropathy. The patient was treated with intravenous methylprednisolone (initial dose of 1,000 mg/d, followed by gradual tapering), resulting in an improvement in limb numbness. After discharge, the patient continued oral prednisone (60 mg/d, with a weekly taper of 5 mg) and VitB12 intramuscular injections. Follow-up at 2 months showed a significant improvement in neurological symptoms, allowing the patient to resume normal daily activities. The RBC count, Hb level, MCV, and MCH gradually became normal (**Table 1**).

## 3 Discussion

Spinal cord SCD is a rare neurodegenerative disorder involving the posterior and lateral columns of the spinal cord and peripheral nerves and is characterized by demyelination of these tracts. The main symptoms include numbness in the lower limbs or all four limbs, a sensation of walking on cotton, gait instability, inability to stand steadily with eyes closed, paralysis, and tingling of the limbs, which are typical early manifestations of spinal cord SCD. CSF examination results are usually normal. MRI of the cervical and upper thoracic spinal cords typically shows linear and punctate lesions with low T1 and high T2 signals. EMG revealed the involvement of both sensory and motor nerves, primarily demyelinating lesions, with some axonal damage. Spinal cord SCD is caused by disturbances in the intake, absorption, binding, transport, or metabolism of VitB12. Other causes of VitB12 deficiency include insufficient dietary intake owing to vegetarianism, gastrectomy, alcoholism with atrophic gastritis, and nitrous oxide abuse. Further investigations to identify the underlying cause and initiate appropriate treatment are essential in patients with spinal cord SCD (6).

In our patient, MRI and EMG findings were consistent with spinal cord SCD. Although the patient’s VitB12 level was within the normal range, his long-term alcohol consumption and laboratory evidence of macrocytic anemia led us to conduct gastroscopy, which revealed CAG.

CAG is typically caused by *Helicobacter pylori* infection, bile reflux, and changes in vasoactive factors and cytokines. It is widely believed that CAG results from the combined effects of multiple factors and that its progression is influenced by long-term genetic changes. Following the diagnosis of CAG, further testing for anti-parietal cell and anti-intrinsic factor antibodies can confirm the presence of autoimmune gastritis, which is crucial in determining the etiology of atrophic gastritis. However, in this case, these antibody tests were not performed because of testing limitations. During the course of the disease, atrophy of the gastric mucosa and intrinsic glands, as well as reduced gastric acid secretion, can severely impair nutrient absorption. One study found that chronic atrophic gastritis is an independent risk factor for VitB12 deficiency in patients with chronic gastritis (7). Although this patient’s VitB12 levels were within the normal range, his macrocytic anemia suggests a possible “functional” deficiency of VitB12 (8). Some studies have indicated that low serum VitB12 levels are not necessary for diagnosing spinal cord SCD. While serum methylmalonic acid (MMA) and homocysteine may be sensitive indicators for assessing VitB12 deficiency, their specificity is relatively low (9).

A case report described a patient with autoimmune polyglandular syndrome type II(APS II) presenting with SCD who also had pernicious anemia, Addison’s disease, and autoimmune thyroid disease (10). This highlights the need to check for other autoimmune diseases when encountering autoimmune-related vitamin B12 deficiencies. In our case, the patient had elevated TSH levels but normal FT3 and FT4, with no signs of skin involvement, endocrine dysfunction, or rheumatic disease. However, thyroid function should be monitored and further tests for anti-TPO and anti-thyroglobulin antibodies are required.

In our case, the positive immune markers were identified as anti-sulfatide IgM and IgG antibodies. High titers of anti-sulfatide antibodies are associated with peripheral nerve disorders (5). In the peripheral nervous system, sulfatide is predominantly found in the non-compact myelin sheaths of Schwann cells, making up 4%–7% of all myelin lipids, and is crucial for the integrity of the myelin sheath (11). When sulfatide is deficient or under attack by an autoimmune response, the paranodal loops and nodes of Ranvier may be disrupted, potentially preventing the myelin sheath from functioning properly (12). Anti-sulfatide antibodies are predominantly found in immune-mediated neuropathies (IMNP) with axonal damage (13). Patients with anti-sulfatide antibodies exhibit higher conduction block rates during nerve conduction studies (14). However, IgM and IgG anti-sulfatide antibodies are not specific markers of particular diseases. The presence of anti-sulfatide antibodies in chronic IMPN remains a contentious issue. Some articles have reported that only 1% of CIDP patients have reactivity to sulfatides (15). Another study emphasized that among patients with CIDP, at least one IgM autoantibody reacts more frequently with GM1, GD1b, and sulfatides (16). Unlike our patient, those with autoantibodies against sulfatides were younger and showed typical clinical manifestations of CIDP (16).

In this case, the presence of positive anti-sulfatide antibodies suggested that the patient had immune-mediated peripheral neuropathy. VitB12 treatment alone was ineffective and required the addition of high-dose steroid therapy. This approach has proven to be effective for patients. However, this case had certain limitations. Due to the variability in the time required for each SCD patient to show a significant response to vitamin B12 replacement therapy, a one-week period may not be sufficient to observe signs of improvement in some cases. Subsequent steroid therapy may have been a confounding variable.

The patient met the criteria for spinal cord SCD, CAG, positive anti-sulfatide antibodies, and macrocytic anemia. While these conditions have been reported individually in the past, to our knowledge, this is the first case in which SCD of the spinal cord overlaps with positivity for anti-sulfatide antibodies. The patient’s symptoms were associated with SCD of the spinal cord and positivity for anti-sulfatide antibodies. Based on the patient’s condition, treatment should include VitB12 and folic acid supplementation as well as immunosuppressive agents for autoimmune diseases. Here, we report this case to enhance our understanding of the relationship between these conditions. We hope that the information from this patient will help other researchers recognize the coexistence of multiple complications related to SCD of the spinal cord and positive anti-sulfatide antibodies and elucidate the overlapping pathogenesis.

## Data Availability

The original contributions presented in the study are included in the article/supplementary material. Further inquiries can be directed to the corresponding author.
